# Factors Associated With Self‐Report Symptom Screening Adherence in Pediatric Cancer Patients

**DOI:** 10.1002/cam4.71053

**Published:** 2025-07-21

**Authors:** L. Lee Dupuis, Emily Vettese, Catherine Aftandilian, Vibhuti Agarwal, Christina Baggott, Scott M. Bradfield, Nicole Crellin‐Parsons, David R. Freyer, Kara M. Kelly, Allison A. King, Wade Kyono, Ramamoorthy Nagasubramanian, Etan Orgel, Michael E. Roth, Farha Sherani, Lolie Yu, Allison C. Grimes, Melissa P. Beauchemin, Lisa M. Klesges, George A. Tomlinson, Lillian Sung

**Affiliations:** ^1^ Program in Child Health Evaluative Sciences, the Hospital for Sick Children Toronto Ontario Canada; ^2^ Department of Pharmacy The Hospital for Sick Children and Leslie dan Faculty of Pharmacy, University of Toronto Toronto Ontario Canada; ^3^ Division of Pediatric Hematology Oncology, Stem Cell Transplant and Regenerative Medicine, Stanford University Palo Alto California USA; ^4^ Nemours Children's Hospital Orlando Florida USA; ^5^ Nemours Children's Health Jacksonville Florida USA; ^6^ Cancer and Blood Disease Institute, Children's Hospital los Angeles Los Angeles California USA; ^7^ Department of Pediatrics Roswell Park Comprehensive Cancer Center, University at Buffalo Jacobs School of Medicine and Biomedical Sciences Buffalo New York USA; ^8^ Division of Public Health Sciences Washington University School of Medicine St. Louis Missouri USA; ^9^ Washington University School of Medicine St. Louis Missouri USA; ^10^ Kapi'olani Medical Center for Women & Children Honolulu Hawaii USA; ^11^ Department of Pediatrics The University of Texas MD Anderson Cancer Center Houston Texas USA; ^12^ Driscoll Children's Hospital, Cancer and Blood Disorders Center and Texas A&M University Corpus Christi Texas USA; ^13^ Louisiana State University Health Sciences Center/Children's Hospital New Orleans Louisiana USA; ^14^ Pediatric Hematology Oncology, University of Texas Health Science Center at san Antonio San Antonio Texas USA; ^15^ Columbia University School of Nursing/Herbert Irving Cancer Center New York New York USA; ^16^ Department of Medicine, Toronto General Hospital Toronto Ontario Canada; ^17^ Division of Haematology/Oncology, the Hospital for Sick Children Toronto Ontario Canada

**Keywords:** adherence, oncology, pediatric, symptom screening

## Abstract

**Introduction:**

Objective was to describe the association between baseline characteristics and the number of Symptom Screening in Pediatrics Tool (SSPedi) assessments completed over an 8‐week period.

**Methods:**

This was a sub‐analysis of a cluster randomized controlled trial among 10 sites that were randomized to the intervention group. Participants were English‐ or Spanish‐speaking pediatric patients 8–18 years of age newly diagnosed with cancer. Participants were prompted to complete SSPedi three times weekly for 8 weeks. The outcome was the number of SSPedi assessments completed during the 8‐week period. Factors associated with the number of assessments were determined using mixed effects Poisson regression.

**Results:**

At the 10 intervention sites, 216 patients were included in the analysis. Among these participants, 129 (59.7%) were male, 112 (51.9%) were white, and 83 (38.4%) were Hispanic. The number of SSPedi assessments was significantly higher for participants 11–14 years (rate ratio (RR) 1.13, 95% confidence interval (CI) 1.02–1.25) and 15–18 years (RR 1.15, 95% CI 1.04–1.27) compared to 8–10 years. Participants completed more SSPedi assessments if they were Asian compared to white (RR 1.27, 95% CI 1.10–1.46), non‐Hispanic compared to Hispanic (RR 1.15, 95% CI 1.04–1.28) and from families with a household income ≥$60,000 (RR 1.12, 95% CI 1.03–1.21). Participants completed fewer SSPedi assessments if they had solid tumors compared to leukemia (RR 0.91, 95% CI 0.84–0.99).

**Conclusion:**

Adherence to three‐times weekly SSPedi varied by age, race, ethnicity, cancer diagnosis, and family income. This information may facilitate interventions to support routine symptom screening in clinical practice.

**Trial Registration:** NCT04614662.

## 1 Introduction

Pediatric patients receiving cancer therapy commonly experience severely bothersome symptoms. Common symptoms include fatigue, changes in hunger, pain, and nausea [1]. These symptoms are rarely documented or treated [2]. To improve symptom control, patients benefit from the implementation of routine symptom screening and clinical practice guideline‐consistent care for symptom management [3]. Symptom screening might be useful as documentation and interventions for symptom management are poor, even when patients experience severely bothersome symptoms [2, 4].

In contrast to adult cancer patients, routine symptom screening is uncommon in pediatric patients. It is uncertain whether pediatric patients would be willing to self‐report symptoms on an ongoing basis. We recently completed a study where patients newly diagnosed with cancer were prompted to complete symptom screening three times weekly for 8 weeks [5, 6]. In this study, symptom screening, when combined with adapted care pathways for symptom management, improved symptom control across most symptoms [5, 6]. Symptom screening could be performed as outpatients or inpatients, mirroring pediatric cancer care settings.

For symptom screening to be effective, pediatric patients must be willing to provide repeated symptom scores. We used the 8‐week longitudinal study as an opportunity to understand participant characteristics associated with the number of Symptom Screening in Pediatrics Tool (SSPedi) assessments submitted. Consequently, the objective was to describe the association between baseline characteristics and the number of SSPedi assessments completed over an 8‐week period.

## 2 Materials and Methods

This was a sub‐analysis of a cluster randomized controlled trial in which 10 sites were randomized to the intervention group and 10 sites were randomized to the control group. This report focuses on participants enrolled at the 10 intervention sites [5]. The study was approved by the Western Institutional Review Board, each participating site's Institutional Review Board, and the Research Ethics Board at The Hospital for Sick Children. Informed consent and assent were obtained from participants and guardians as appropriate. The study was registered with clinicaltrials.gov (NCT04614662).

### 2.1 Eligibility

Participants were English‐ or Spanish‐speaking pediatric cancer patients 8–18 years of age with a plan for any cancer treatment. They had to have been newly diagnosed with cancer or have started cancer treatment within the 4 weeks prior to enrollment. Those with cognitive disabilities or visual impairments precluding routine symptom screening were excluded.

### 2.2 Study Procedures

Intervention sites adapted symptom management care pathway templates based upon clinical practice guidelines before the site was activated to participant enrollment. Once activated, patients were approached in the inpatient setting, outpatient setting, or remotely. Participants could consent to provide patient‐reported outcomes or to chart review only. For those who agreed to provide patient‐reported outcomes, demographic characteristics were obtained from the participant, family, or health record. Participants were set up to complete SSPedi on their personal device, which could be a phone, tablet, or computer. If a device was not available, a tablet was loaned to them for the study duration. SSPedi is a self‐report symptom screening and assessment tool that is reliable, valid, and responsive to change in pediatric patients receiving cancer treatments 8–18 years of age [7]. SSPedi is available in English, Spanish [8, 9] and French [10] although only the English and Spanish versions were used in this study. SSPedi completion requires about 1 min to complete.

We used a web application named Supportive care Prioritization, Assessment and Recommendations for Kids (SPARK) to enable participants to receive reminders, allow participants and healthcare professionals to track symptoms, and provide email alerts to the healthcare team [11, 12]. Participants received reminders to complete symptom screening by text or email according to their preference. They also could enable their guardian to receive a concurrent reminder to facilitate pediatric patient self‐report. They were reminded to complete symptom screening three times weekly for 8 weeks, but they could complete SSPedi on their own at any time outside of the reminders. Participants could choose the days of the week and the times of reminders. These days and times could be changed during the eight‐week period with the assistance of the local research team. If participants did not complete any SSPedi assessments after 1–2 weeks, the local research team reached out to the participant to identify if they were experiencing challenges such as forgetting their password. Each time participants completed SSPedi, an email alert was sent to their healthcare team if they reported at least one symptom that was severely bothersome, defined as a score of 3 or 4 on a 5‐point Likert scale ranging from 0 to 4. The healthcare team recipients were chosen by the site, and the recipients could change over time for individual participants.

To support symptom management, implementation materials such as posters, pens, and badges with QR codes linked to the site‐specific care pathways were created. The email alert based on severely bothersome symptoms included a link to these care pathways.

### 2.3 Statistical Analysis

The outcome for this analysis was the number of SSPedi assessments completed during the eight‐week period. We assessed the dependence of the number of SSPedi assessments completed on participant and guardian characteristics using a mixed effects Poisson regression model with a random effect for site. The model was fitted using maximum likelihood and included six participant characteristics (sex, age group, race, ethnicity, preferred language, and cancer diagnosis group) and three guardian characteristics (marital status, guardian employment status and high annual household income). Rate ratios (RR) for the number of SSPedi assessments completed per week were estimated for each level of a characteristic compared to its reference level, along with 95% confidence intervals (CI) and Wald‐test P values. Global tests of significance of each multi‐level characteristic were obtained from likelihood ratio tests comparing the full model (with all nine characteristics) to a model omitting that characteristic. As there were only 10 intervention sites, we did not evaluate site characteristics in the model. However, we described between‐site variability in average SSPedi adherence after consideration for site and adjustment for patient characteristics.

As a post hoc analysis, we also evaluated the impact of baseline SSPedi score quartile on adherence using the same mixed effects Poisson regression model. Tests of significance were two‐sided, and statistical significance was defined as *p* < 0.05. Statistical analysis was conducted using R 4.3.2.

## 3 Results

As previously reported, 217 participants were enrolled at intervention sites and agreed to provide patient‐reported outcomes [5, 6]. One participant came off the study prior to completing study observations and thus, 216 participants were included in this analysis [5]. Table 1 shows the demographic distribution of the cohort; 129 (59.7%) were male, 112 (51.9%) were white, and 83 (38.4%) were Hispanic or Latino.

**TABLE 1:** Mean number of SSPedi assessments and their association with baseline characteristics (*N* = 216).

Characteristic	*N*	Mean (SD)^a^	RR^a^ (95% CI)	*p*	
				vs. Reference	Group^a^
Sex					
Female	87	18.1 (8.0)	REF		NA
Male	129	17.6 (8.5)	1.00 (0.93, 1.07)	1.00	
Age group in years					
8–10	33	16.8 (8.4)	REF	—	0.02
11–14	76	17.8 (8.0)	1.13 (1.02, 1.25)	0.02	
15–18	107	18.1 (8.5)	1.15 (1.04, 1.27)	0.008	
Race					
American Indian or Alaska Native	2	14.5 (17.7)	1.13 (0.77, 1.67)	0.52	< 0.001
Asian	13	21.0 (7.5)	1.27 (1.10, 1.46)	< 0.001	
Black or African American	13	16.2 (9.9)	0.92 (0.78, 1.07)	0.27	
Native Hawaiian or Other Pacific Islander	14	15.8 (6.3)	0.92 (0.77, 1.11)	0.39	
White	112	17.6 (8.4)	REF	—	
Unknown/prefer not to say	40	18.0 (7.6)	1.28 (1.13, 1.45)	< 0.001	
More than one	22	19.2 (9.0)	1.14 (1.01, 1.29)	0.03	
Ethnicity					
Hispanic or Latino	83	17.0 (8.5)	REF	—	0.02
Not Hispanic or Latino	122	18.4 (8.1)	1.15 (1.04, 1.28)	0.009	
Unknown/prefer not to say	11	17.5 (9.3)	1.01 (0.86, 1.19)	0.90	
Preferred language for patient‐reported outcomes					
English	203	17.9 (8.3)	1.12 (0.96, 1.31)	0.15	NA
Spanish	13	16.2 (7.5)	REF	—	
Cancer diagnosis					
Leukemia	87	18.2 (8.1)	REF	—	0.03
Lymphoma	48	17.5 (8.5)	0.92 (0.84, 1.01)	0.08	
Solid tumor	73	17.0 (8.4)	0.91 (0.84, 0.99)	0.02	
Brain tumor	8	22.2 (8.1)	1.09 (0.91, 1.30)	0.37	
Family composition					
Guardian married	137	18.2 (8.3)	REF		NA
Guardian not married	79	17.1 (8.2)	0.98 (0.91, 1.05)	0.51	
Guardian employment					
Full time or part time	123	17.8 (8.2)	0.93 (0.86, 1.00)	0.05	NA
Other	93	17.9 (8.4)	REF	—	
Annual household income					
≥ $60,000	77	19.2 (7.2)	1.12 (1.03, 1.21)	0.007	NA
< $60,000	139	17.0 (8.7)	REF	—	

Abbreviations: CI, confidence interval; NA, not applicable; RR, rate ratio; SD, standard deviation; SSPedi, Symptom Screening in Pediatrics Tool.

^a^ Mean number of assessments was only descriptive. Statistical association was determined using mixed effects Poisson regression adjusting for site as a random effect. *p* values for multi‐level characteristics were calculated from likelihood ratio tests.

**FIGURE 1:**
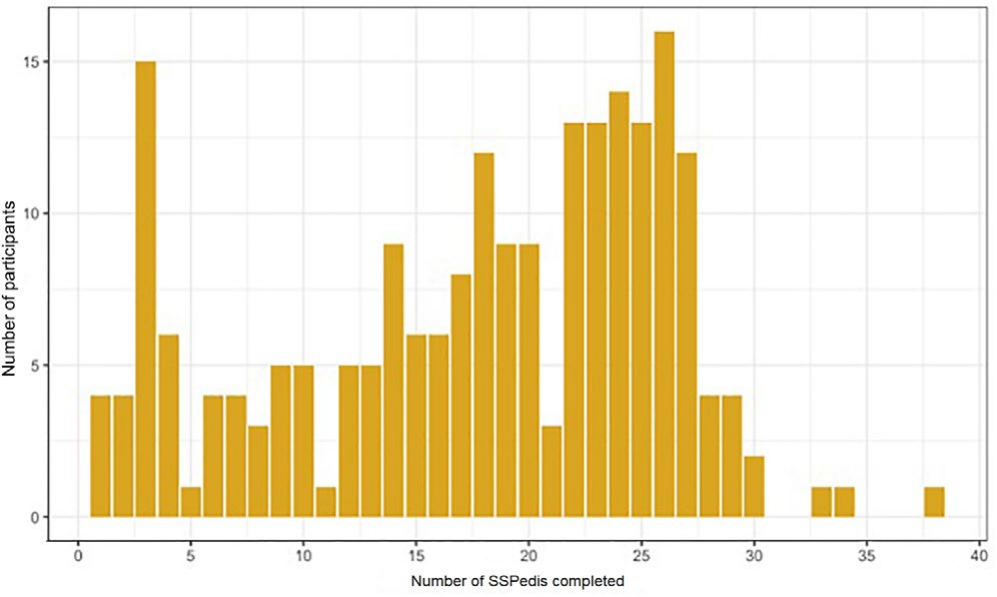
Bar graph showing number of SSPedi assessments completed. SSPedi, Symptom Screening in Pediatrics Tool.

Figure 1 shows a histogram describing the SSPedi assessment count distribution. Table 1 shows that the number of SSPedi assessments was significantly higher for participants 11–14 years (RR 1.13, 95% CI 1.02–1.25) and 15–18 years (RR 1.15, 95% CI 1.04–1.27) compared to 8–10 years. Participants completed more SSPedi assessments if they were Asian compared to white (RR 1.27, 95% CI 1.10–1.46), non‐Hispanic compared to Hispanic (RR 1.15, 95% CI 1.04–1.28), and from families with a household income ≥$60,000 (RR 1.12, 95% CI 1.03–1.21). Participants completed fewer SSPedi assessments if they had solid tumors compared to leukemia (RR 0.91, 95% CI 0.84–0.99). All multi‐level characteristics containing at least one statistically significant comparison to their reference group had statistically significant likelihood ratio tests. Table 1 also shows the mean number of SSPedi assessments for descriptive purposes.

**FIGURE 2:**
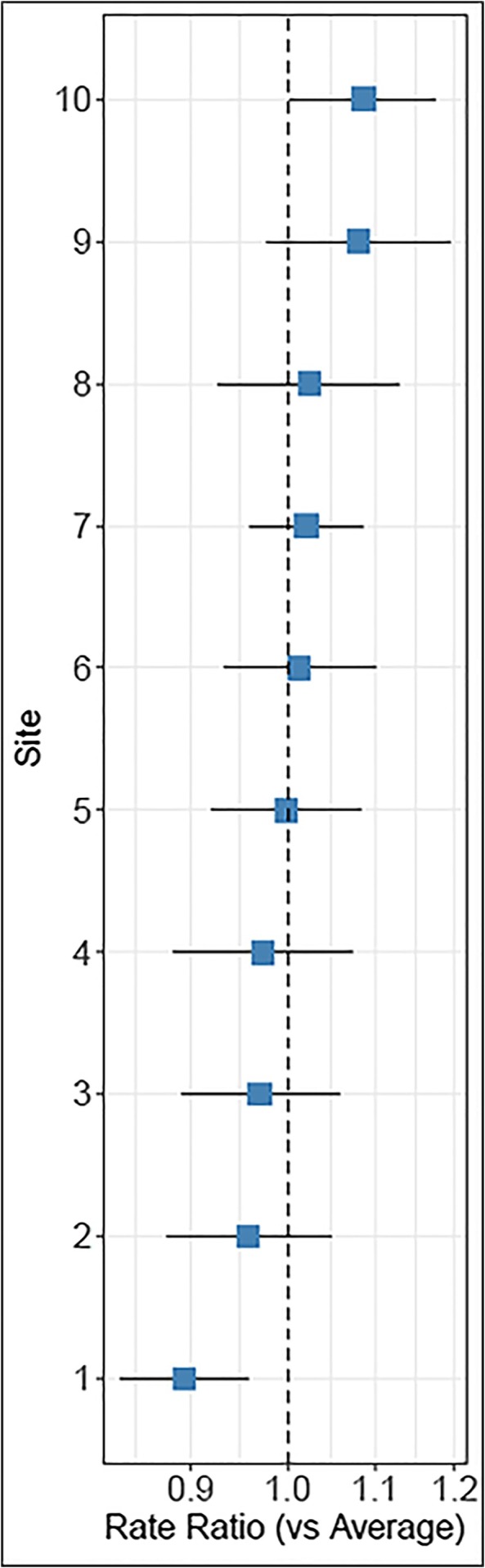
Number of SSPedi assessments completed by site compared to the average. SSPedi, Symptom Screening in Pediatrics Tool.

**FIGURE 3:**
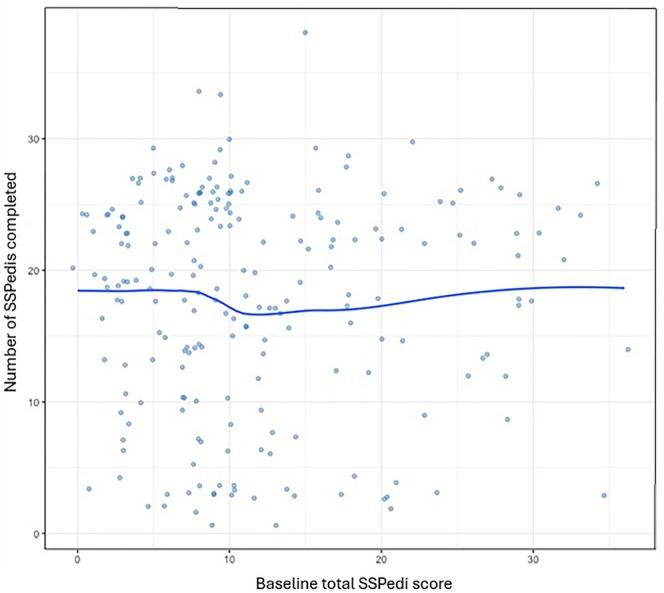
Relationship between number of SSPedi assessments and Baseline Total SSPedi Score. SSPedi, Symptom Screening in Pediatrics Tool.

Figure 2 shows substantial between‐site variability in average SSPedi adherence after consideration of site and adjustment for patient characteristics. Figure 3 shows the relationship between baseline SSPedi score on adherence. In the post hoc mixed effects Poisson regression analysis, baseline SSPedi quartile was not significantly associated with adherence (*p* = 0.38).

## 4 Discussion

We found that age, race, ethnicity, cancer diagnosis, and family income explained variability in SSPedi adherence over 8 weeks. More specifically, those with younger ages, Hispanic or Latino ethnicity, solid tumors, and households with lower family income completed significantly fewer SSPedi assessments. Asian participants completed significantly more SSPedi assessments compared to white participants.

Some studies have demonstrated high adherence rates to routine patient‐reported outcome monitoring among adult cohorts [13]. Factors identified to be associated with non‐adherence have included younger age, more comorbidities, absence of chemotherapy, and low physical functioning score at baseline [14]. A study of longitudinal symptom screening in adults showed that adherence decreased from 72% to 52% over a 10‐week period [15]. Nonetheless, a systematic review of chronic conditions outside of cancer was unable to conclude that any factor was consistently associated with non‐adherence [16].

In contrast to these reports, very few studies have evaluated factors associated with adherence in pediatric populations. Pediatric patients are expected to have additional challenges to symptom screening adherence compared to adult patients. Our previous study highlighted the importance of engaging with guardians to enable their child to self‐report SSPedi, particularly for younger children [17]. Thus, the finding that younger children reported fewer SSPedi assessments is not surprising. It is likely that independent completion of repeated SSPedi assessments will continue to be challenging among the youngest patients. It is for this reason that we created a novel structured dyadic approach to symptom screening that allows guardians to support their child while ensuring the child voices their perspectives first [18, 19, 20]. This instrument, named co‐SSPedi, is reliable, valid, and responsive to change [21].

It is also interesting that we showed that race, ethnicity, and household income were associated with SSPedi adherence. This finding suggests that specific interventions may be required to promote adherence among some patient sub‐groups. Such interventions will help to mitigate widening disparities that will arise without purposeful efforts. It is also interesting that there was heterogeneity in SSPedi adherence across sites. As this analysis adjusted for patient factors, it is more likely that this variability reflects site procedures, values, and possibly resources. While the number of sites did not permit evaluation of site characteristics on SSPedi adherence, interventions at the site level may also be helpful.

The increased adherence among leukemia participants compared to solid tumor participants may be related to increased inpatient stays and more frequent contact with the healthcare system in general for leukemia patients. While not statistically significant, it is interesting that brain tumor patients had qualitatively higher rates of SSPedi assessment completion. This observation may be related to symptom burden among this group. The low number of brain tumor patients may have been related to some of these patients receiving their primary care from non‐oncologists such as neurosurgeons. Nonetheless, the cancer group approach is likely too broad, and future work could evaluate SSPedi assessment adherence by specific diagnoses and treatment protocols.

A strength of this report is the ability to leverage a large database of longitudinal pediatric patient self‐report symptom screening assessments. Another strength is the multi‐center nature and the heterogeneity of the cohort. However, a limitation of the study is that we only instituted symptom screening for 8 weeks. Also, potentially important explanatory variables were not collected or were not available to the lead site. Future research should explicitly collect variables such as guardian receipt of reminders, access to information technology, and additional socioeconomic variables. Time‐dependent variables such as treatment intensity, concurrent chemotherapy, and inpatient or outpatient status could be included in this evaluation. Qualitative evaluation, which was not included as part of this study, might be particularly useful to identify if events such as emergency visits might increase or decrease SSPedi adherence.

## 5 Conclusion

Adherence to three‐times weekly SSPedi varied by age, race, ethnicity, and family income. This information may facilitate interventions to support routine symptom screening in clinical practice. Some sub‐populations might benefit from additional support, such as structured outreach to improve adherence to routine symptom screening.

## Author Contributions

**L. Lee Dupuis:** conceptualization (equal), writing – original draft (equal), writing – review and editing (equal). **Emily Vettese:** conceptualization (equal), project administration (equal), writing – review and editing (equal). **Catherine Aftandilian:** conceptualization (equal), writing – review and editing (equal). **Vibhuti Agarwal:** conceptualization (equal), investigation (equal), writing – review and editing (equal). **Christina Baggott:** conceptualization (equal), investigation (equal), writing – review and editing (equal). **Scott M. Bradfield:** conceptualization (equal), writing – review and editing (equal). **Nicole Crellin‐Parsons:** conceptualization (equal), project administration (equal), writing – review and editing (equal). **David R. Freyer:** conceptualization (equal), investigation (equal), writing – review and editing (equal). **Kara M. Kelly:** conceptualization (equal), investigation (equal), writing – review and editing (equal). **Allison A. King:** conceptualization (equal), writing – review and editing (equal). **Wade Kyono:** conceptualization (equal), investigation (equal), writing – review and editing (equal). **Ramamoorthy Nagasubramanian:** conceptualization (equal), writing – review and editing (equal). **Etan Orgel:** conceptualization (equal), investigation (equal), writing – review and editing (equal). **Michael E. Roth:** conceptualization (equal), investigation (equal), writing – review and editing (equal). **Farha Sherani:** conceptualization (equal), investigation (equal), writing – review and editing (equal). **Lolie Yu:** conceptualization (equal), investigation (equal), writing – review and editing (equal). **Allison C. Grimes:** conceptualization (equal), writing – review and editing (equal). **Melissa P. Beauchemin:** conceptualization (equal), investigation (equal), writing – review and editing (equal). **Lisa M. Klesges:** conceptualization (equal), writing – review and editing (equal). **George A. Tomlinson:** conceptualization (equal), formal analysis (equal), writing – original draft (equal), writing – review and editing (equal). **Lillian Sung:** conceptualization (lead), writing – original draft (equal), writing – review and editing (equal).

## Conflicts of Interest

The following authors declare conflicts of interest: E.O. consults with Jazz Pharmaceuticals.

## Data Availability

The data that support the findings of this study are openly available at https://clinicaltrials.gov/.
